# Understanding Global Health Inequality and Inequity: Causes, Consequences, and the Path Toward Justice in Healthcare

**DOI:** 10.1002/puh2.70156

**Published:** 2025-10-26

**Authors:** Yusuf Hared Abdi, Mohamed Sharif Abdi, Sharmake Gaiye Bashir, Naima Ibrahim Ahmed, Yakub Burhan Abdullahi

**Affiliations:** ^1^ Department of Environmental Science Faculty of Geoscience & Environment Hormuud University Mogadishu Somalia; ^2^ De Martino Public Hospital Ministry of Health and Human Services, Federal Government of Somalia Mogadishu Somalia

**Keywords:** global health, health inequality, health inequity

## Abstract

**Background:**

Health inequality and inequity persist as pressing global challenges that disproportionately affect populations in low‐ and middle‐income countries. These disparities are driven by complex and interrelated factors, including socioeconomic deprivation, educational barriers, commercial determinants of health, inadequate governance structures, and systemic failures in resource allocation.

**Objectives:**

This study seeks to critically examine the multifactorial causes of global health inequality and inequity, assess their wide‐ranging consequences on population health, and propose ethically grounded strategies for promoting equity and justice within global healthcare systems.

**Methods:**

A rapid review approach was used to examine global health inequality and inequity. Searches were conducted in PubMed, Google Scholar, and Research Rabbit using key terms such as “global health inequality,” “health equity in global health policy,” and “healthcare disparities across countries.” The literature was selected on the basis of its relevance to structural determinants and systemic patterns of health disparities across countries with varying income levels. The analysis identified recurring themes related to the drivers of inequity and potential pathways toward healthcare systems while acknowledging the limitations inherent in rapid review methodologies, including possible language and selection biases.

**Results:**

The findings highlight that structural determinants, such as poverty, educational and gender disparities, environmental degradation, conflict, and unequal allocation of health resources, serve as primary contributors to global health inequities. These factors result in disproportionate disease burden, limited access to essential services, and increased mortality rates in disadvantaged populations.

**Conclusion:**

Addressing global health inequity requires a transformative, ethically grounded framework that emphasizes justice, solidarity, and equity. Key actions include redistributive policy reforms, strengthened health systems in resource‐limited settings, and the decolonization of global health governance. These measures are fundamental for realizing the right to health and achieving meaningful global health justice.

## Graphical abstract

This graphical abstract illustrates the central theme of global health inequality and inequity. On the left, structural determinants, such as poverty, inadequate education, commercial and environmental factors, conflict, and weak governance, are depicted as key drivers. These lead to unequal health outcomes globally, including disparities in vaccine access, elevated morbidity and mortality, and gaps in healthcare services, as shown in the center. On the right, targeted solutions are highlighted: rights‐based policy frameworks, strengthened health systems, digital health inclusion, environmental justice, and equitable workforce distribution all contributing to the ultimate goal of health equity and justice for all populations.

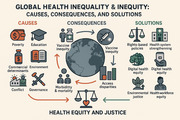

## 1 Introduction

Health inequality and inequity are critical constructs in the discourse on global public health, underpinning the uneven distribution of health outcomes and access to healthcare services across and within countries [1]. Health inequality refers to observable differences in health status or the allocation of health resources among distinct population groups, often measured through indicators such as life expectancy, disease burden, and service utilization [2]. These disparities frequently reflect social stratification based on income, education, sex, ethnicity, geography, and other demographic determinants [2, 3].

Although health inequalities describe measurable differences, they are defined by their moral dimension, denoting disparities that are avoidable, unjust, and unnecessary [4, 5]. Health inequities typically result from systematic social disadvantages, discrimination, and exclusion, which disproportionately affect marginalized populations and contribute to cumulative health disadvantages [5]. As these inequities are socially constructed, they are by nature potentially remediable through intentional policy and structural change [6]. The concept of health equity is embedded in the broader framework of human rights, which calls for the elimination of unfair and avoidable differences in health and access to care as well as equitable health financing and distribution of resources [7].

The persistence of health inequalities is a global concern. Empirical evidence illustrates that, even within high‐income settings, socially and economically marginalized groups experience disproportionately poor health outcomes [7, 8]. In low‐ and middle‐income countries (LMICs), regional disparities in healthcare access and service utilization, particularly in maternal and child health, remain stark, with Asia and Africa exhibiting the most significant intraregional inequalities [9]. Furthermore, the global burden of diseases spanning communicable and non‐communicable diseases (NCDs) and injuries remains inequitably distributed, reflecting entrenched systemic inequities [3]. Structural determinants, such as globalization and neoliberal economic policies, have exacerbated these disparities by shaping the distribution of power, resources, and opportunities in ways that reinforce disadvantages [10].

Global health inequity is an issue related to vaccine distribution during the COVID‐19 pandemic [7]. High‐income countries (HICs) have significantly higher vaccination rates than low‐income countries by the end of 2021 [6, 11]. Specifically, 64.1% of individuals in HICs had received at least one dose, whereas only 5.4% of people in low‐income countries had received this dose. Moreover, nearly 75% of the population in HICs was fully vaccinated, compared to only 21% in low‐income countries [6]. Beyond these numbers, the distribution of the most effective vaccines, namely, Pfizer–BioNTech and Moderna, was heavily skewed in favor of wealthy nations, whereas LMICs primarily received the Oxford–AstraZeneca vaccine despite growing evidence of efficacy disparities by late 2021 [10, 12]. This inequity was exacerbated by vaccine nationalism, as HICs secured substantial supplies through advance purchase agreements, effectively monopolizing production and leaving LMICs reliant on delayed or surplus supplies [6, 10]. Such patterns reinforce historical structural inequalities, demonstrating that global market forces and geopolitical interests often override public health equity [6].

Addressing global health inequality and inequity is an urgent and ethical imperative. Despite overall improvements in health outcomes globally, socioeconomic health disparities have persisted and widened in some contexts [13]. The 2015 United Nations Sustainable Development Goals (SDGs) underscore the importance of equity as a cross‐cutting principle, particularly in the goal of ensuring healthy lives and promoting well‐being for all at all ages [5, 13]. The COVID‐19 pandemic has further exposed the depth of existing health inequities, disproportionately affecting vulnerable populations, including people with disabilities, who face higher risks of infection, severe illness, and mortality [14]. Tackling these inequities requires an in‐depth understanding of their structural roots and transformative change at the individual, institutional, and policy levels [1]. Failure to do so not only perpetuates injustice and human suffering but also hinders global efforts toward sustainable and inclusive development. Therefore, a paradigm shift is an essential one that emphasizes social justice, equitable redistribution of power and resources, and inclusive health governance to achieve health equity for all populations [15].

This article examines the structural causes, consequences, and solutions of health inequities globally, emphasizing justice‐oriented policy reform.

## 2 Methods

This narrative review was conducted to critically examine global health inequality and inequity, emphasizing a comprehensive synthesis of theoretical frameworks and empirical evidence. A selective literature search was performed across PubMed, Google Scholar, and Research Rabbit databases using search terms including “global health inequality,” “health equity,” “commercial determinants of health (CDoH),” “intellectual property rights health,” “neglected tropical diseases (NTDs),” “digital health divide,” “environmental degradation health,” “conflict health inequality,” “rights‐based approaches health,” and “historical injustice global health.” The search strategy employed both individual and combined terms to capture the multidimensional nature of global health inequities. Literature selection prioritized peer‐reviewed articles, reports from international organizations (WHO, UN, World Bank), and gray literature that addressed structural determinants, systemic patterns of health disparities, and theoretical frameworks. This narrative approach allowed for critical synthesis and theoretical engagement with complex, interconnected factors driving global health inequities while acknowledging the inherent limitations of non‐systematic methodologies, including potential selection bias and incomplete coverage of all relevant literature.

### 2.1 Conceptual Framework: Inequality vs. Inequity

Health inequality and health inequity, although often used interchangeably, represent distinct conceptual frameworks that are essential for understanding global health disparities [7]. Health inequality refers to measurable differences in health outcomes or resource distribution between populations, which may arise from natural variation, personal choices, or unavoidable circumstances, such as genetic factors or age‐related changes [13]. By contrast, health inequity denotes unfair, avoidable, and systematic differences rooted in social injustice, discrimination, and structural barriers that prevent equal access to health‐determining resources and opportunities [16].

This distinction is critical in policy formulation and intervention design [17, 18]. Under what conditions are health inequalities considered unjust? Drawing from Whitehead's seminal work and subsequent theoretical developments, health differences become inequitable when they are (1) systematically associated with social disadvantage, (2) modifiable through reasonable policy interventions, (3) arise from unfair distribution of social determinants of health, and (4) violate the principles of human dignity and rights [19, 20]. This framework guided our analysis of global health disparities, focusing on addressable injustices rather than inevitable variations [19].

### 2.2 CDoH and Global Inequity

A critical gap in traditional global health discourse has been insufficient attention paid to CDoH—the ways in which commercial actors and systems influence health outcomes and equity [21, 22]. Commercial determinants encompass the practices, products, and power dynamics of private‐sector entities that affect population health, often disproportionately affecting vulnerable populations [21, 23]. These include the marketing strategies of unhealthy commodity industries (tobacco, alcohol, and ultra‐processed foods), pharmaceutical pricing mechanisms that limit access to essential medicines, and corporate influence on health policy through lobbying and regulatory capture [24, 25].

The COVID‐19 pandemic has illustrated how commercial interests can override public health equity [26, 27]. Intellectual property rights regimes, although intended to incentivize innovation, created barriers to equitable vaccine access when pharmaceutical companies prioritized profit maximization over global health security [26]. The concentration of vaccine production in HICs, combined with aggressive patent protection, exemplifies how commercial determinants can perpetuate global health inequities [28]. Trade agreements, particularly those strengthening intellectual property protection, often constrain LMICs’ abilities to produce or import affordable generic medicines and health technologies [29, 30].

### 2.3 NTDs and Health Equity

NTDs represent paradigmatic examples of how global health inequities manifest [31]. Affecting over one billion people, predominantly in low‐income settings, NTDs persist not due to lack of medical knowledge, but because they affect populations with limited economic and political power [31, 32]. Market failure in NTD R&D reflects broader patterns, where commercial incentives align poorly with global health needs [31]. These diseases perpetuate cycles of poverty and social exclusion, particularly affecting women, children, and marginalized communities who bear the greatest burden yet have the least voice in a health priority setting [31, 32].

The WHO's neglected tropical disease roadmap represents an important framework for addressing these inequities, emphasizing cross‐sectoral collaboration, community engagement, and integrated interventions [31]. However, sustainable progress requires addressing the underlying social determinants, strengthening health systems, and ensuring meaningful participation of affected communities in program design and implementation [31, 32].

### 2.4 Digital Health Divide and Access Inequities

The rapid digitization of healthcare has created new dimensions of health inequity through the digital health divide [33, 34]. Although digital health technologies offer unprecedented opportunities to improve access to and quality of care, they often exacerbate existing disparities by privileging populations with greater digital literacy, technological access, and economic resources [33, 34]. The digital divide operates at multiple levels: access to devices and internet connectivity (first‐level divide), digital skills and health literacy (second‐level divide), and differential benefits from digital health interventions (third‐level divide) [33, 34].

In LMICs, inadequate digital infrastructure, limited technological access, and low digital literacy create barriers to telehealth services, electronic health records, and digital health information [33, 34]. Even when digital health solutions are available, they may not be culturally appropriate or linguistically accessible to a diverse population. Addressing the digital health divide requires targeted investments in infrastructure, digital literacy programs, and inclusive design principles that prioritize equity [33, 34].

### 2.5 Health Workforce Distribution and Global Inequities

Global health workforce distribution represents a fundamental driver of health inequities, with a six‐fold difference in health worker density between high‐income and low‐income countries [35, 36]. This inequitable distribution results from complex factors including limited health worker production capacity in low‐resource settings, inadequate working conditions and compensation, and migration patterns that facilitate “brain drain” from countries that most need health workers [35, 37]. The COVID‐19 pandemic highlighted how health workforce shortages disproportionately affect vulnerable populations’ access to care [37]. In sub‐Saharan Africa, where the burden of communicable diseases remains high and NCDs are rising, health worker shortages constrain system capacity to address population health needs effectively [38, 39]. Addressing workforce inequities requires coordinated global action, including ethical recruitment practices, investment in health worker education and retention in low‐resource settings, and the recognition of diverse health worker roles, including community health workers [37, 40].

### 2.6 Environmental Degradation, Conflict, and Health Inequities

Environmental degradation and conflict represent interconnected drivers of global health inequities that disproportionately affect populations in LMICs [41, 42]. Climate change, environmental pollution, and resource scarcity contribute to health inequities through multiple pathways: direct health impacts from extreme weather events and pollution exposure; indirect effects through food insecurity and forced migration; and exacerbation of existing social vulnerabilities [41, 42].

Conflict and environmental degradation often create vicious cycles, where resource scarcity contributes to social tensions, whereas conflict further degrades environmental conditions. In fragile and conflict‐affected states, health systems are particularly vulnerable to disruption, leaving populations with no access to essential services during critical periods. Women, children, and marginalized groups bear disproportionate burdens during conflicts and experience increased risks of gender‐based violence, malnutrition, and communicable disease outbreaks [42, 43].

Academically, health inequality often has a bivariate connotation, investigating whether people with “better” health also have a high income or material well‐being [4]. Health inequities, on the other hand, are disparities that are unnecessary, avoidable, unfair, unjust, and systematically disadvantage socially disadvantaged groups with respect to their health [4].

HICs such as the United States often exhibit health disparities across different socioeconomic, racial, and ethnic groups, even with a relatively well‐resourced healthcare system [44]. For instance, racial and ethnic minority groups with disabilities in the United States may experience disparities in health, insurance coverage, and health service use compared to white individuals with disabilities [14]. This finding can be linked to factors such as income inequality [45]. These factors operate at different levels individual, community, national, and global shaping health outcomes through complex, interrelated pathways. Although these are significant and often inequitable inequalities, the fundamental health system infrastructure might be more developed than in many low‐income countries [44]. Low‐income countries often face systemic inequities in healthcare access and outcomes owing to widespread poverty, limited resources, and weaker health systems [46]. The WHO's work highlights that LMICs account for almost all pregnancy‐related mortalities, largely preventable through adequate utilization of essential maternal healthcare services but hindered by policy‐ and capacity‐related barriers and widespread inequality [9]. For example, in some low‐income countries, more than three‐quarters of women may remain deprived of antenatal care (ANC) and skilled birth assistance (SBA) services [9]. These inequities are often deeply embedded in social, economic, and political structures, limiting access to basic necessities, such as safe water, sanitation, and healthcare for large segments of the population [47]. The inverse care law describes how those most in need of health care are least likely to receive it, often compounded by their socioeconomic disadvantage [8]. In essence, although a high‐income country such as the United States might have disparities arising from factors such as unequal access to insurance or discriminatory practices within the system, a low‐income country might have systemic inequities where the entire health system struggles to provide basic care to a significant portion of its population due to resource constraints and structural barriers [14]. Monitoring these inequalities and understanding whether they are inequitable are crucial for developing effective public health policies aimed at promoting health equity [5, 44].

**FIGURE 1:**
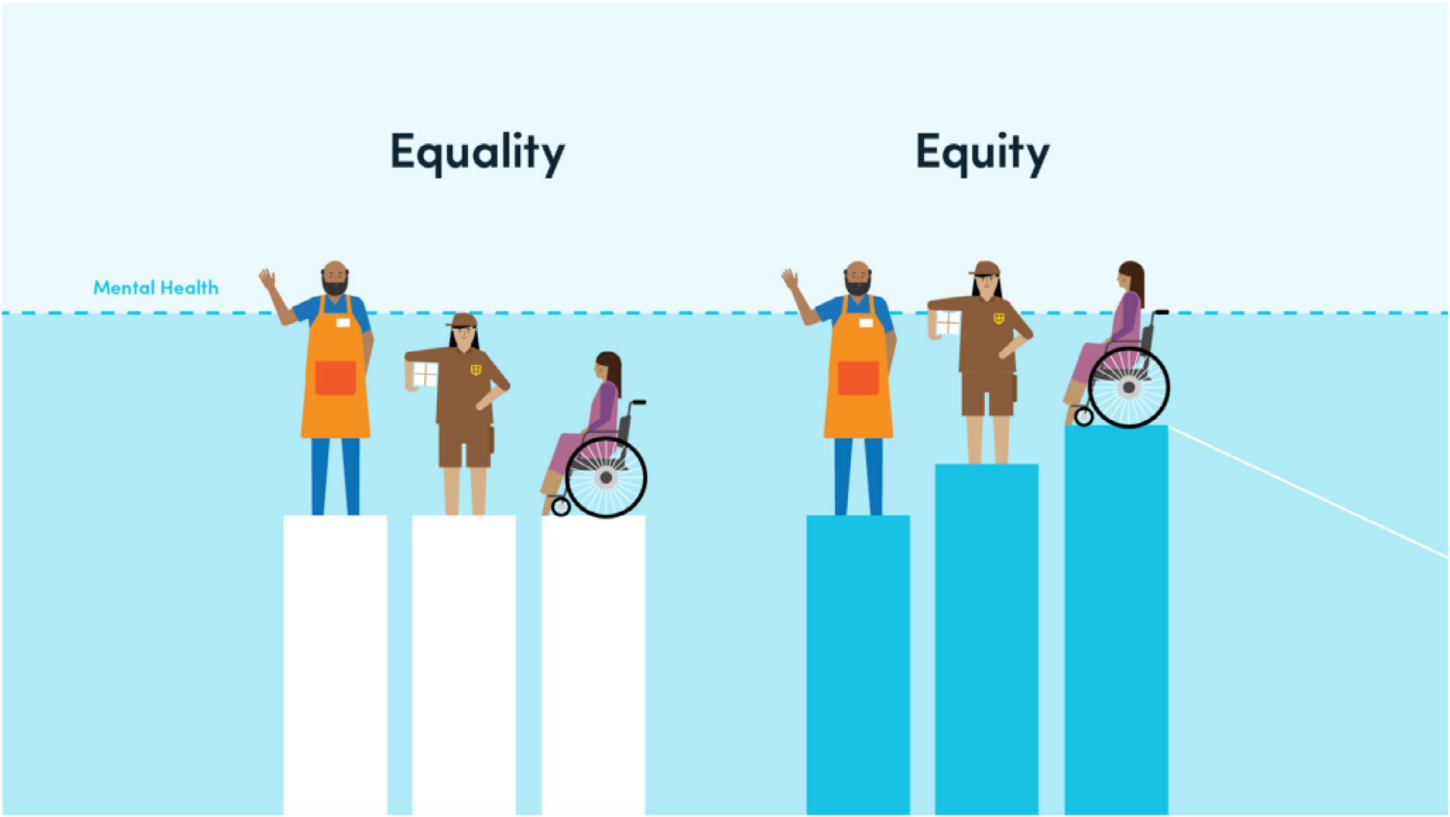
Visual comparison of equality and equity in mental health access.

This observation (Figure 1) provides a comparative visual representation of the concepts of equality and equity in the context of mental health access and support. On the left side labeled “Equality,” individuals are given identical support platforms regardless of their differing needs or circumstances. Although each person receives the same assistance, the outcome remains unequal, particularly evident for individuals using a wheelchair who cannot reach the same level of mental well‐being as others.

Conversely, the right side labeled “Equity” illustrates an approach where support is tailored according to individual needs, resulting in a more balanced outcome. Here, the wheelchair user is provided with enhanced support, allowing them to reach the same mental health threshold as their peers. This distinction emphasizes that equitable interventions—those that consider and respond to diverse individual conditions—are more effective in achieving fair and just outcomes in public health, particularly mental health.

This image underscores the importance of equity over equality in health systems and policy design, highlighting how equal treatment does not necessarily yield equal results when the starting conditions are inherently unequal.

## 3 Determinants of Global Health Inequality and Inequity

Global health inequality and inequity represent profound challenges in the pursuit of well‐being, manifesting as unfair and avoidable differences in health status and access to healthcare across and within countries [8]. These disparities are shaped by a complex interplay of interconnected determinants that operate at individual, community, national, and global levels.

Socioeconomic status, encompassing factors such as poverty and income gaps, is a fundamental determinant of global health inequality [8, 48]. Inequities in power, money, and resources drive disparities in the conditions under which people are born, live, work, and age [49]. The paradoxical “development gap” between opulence and deprivation results in staggering global inequalities, excluding many from mainstream economic activity and consequently impacting health outcomes [47]. Indeed, lower socioeconomic status is consistently associated with poorer health, reduced access to quality healthcare, and an increased risk of both communicable diseases and NCDs [8, 48]. Income inequality has been shown to have more harmful effects on health in poor countries than in rich ones, indicating a global gradient in this relationship [45].

Education and health literacy are critical factors that influence an individual's ability to navigate the healthcare system, adopt healthy behaviors, and understand health information [50]. Limited health literacy, particularly among vulnerable populations, such as migrants, can contribute to inequity in health and healthcare, leading to poorer communication with healthcare providers and reduced benefits from health interventions [51]. Furthermore, lower education levels are often intertwined with lower socioeconomic status, compounding health disadvantages [14].

Gender and minority discrimination are pervasive social determinants that contribute significantly to global health inequity [5]. Discrimination based on gender, race, ethnicity, and other minority statuses can lead to systemic disadvantages in access to resources, including healthcare, education, and employment, ultimately impacting health outcomes [50, 52]. Health inequity researchers emphasize that systems of marginalization, such as racism and sexism, are at the core of racial and non‐racial health inequities, operating at the macro, meso, and micro levels [52]. Addressing health inequalities requires confronting the centrality of subordination favored by policies, practices, norms, and cultures that disadvantage certain social groups [6].

Geographic barriers, particularly the divide between rural and urban areas, present significant obstacles in achieving health equity [50]. Rural populations often face challenges related to the distance to healthcare facilities, limited availability of healthcare professionals, and poorer infrastructure, leading to reduced access to timely and quality care [50, 53]. These disparities can result in poorer health outcomes for individuals living in remote or underserved areas compared with their urban counterparts [50].

Health system capacity and coverage are crucial for ensuring equitable access to healthcare services [8]. Inadequate health system capacity, characterized by limited human resources, lack of training, and skill gaps among healthcare workers, can hinder access to care, particularly for marginalized populations such as persons with disabilities who may require longer or more frequent sessions and reasonable accommodations [14]. Universal health coverage (UHC), aimed at providing all people with access to needed health services without financial hardship, is a stated objective for many governments and international organizations; however, achieving equitable UHC requires addressing disparities in access, utilization, and quality of care [8, 54].

Global health inequality and inequity are multifaceted issues driven by a complex web of interconnected determinants. Addressing these disparities and moving toward justice in healthcare necessitates comprehensive and coordinated efforts that tackle socioeconomic disadvantages, improve education and health literacy, combat discrimination, overcome geographic barriers, strengthen health system capacity, and promote stable and equitable political environments. Recognizing the interplay between these factors is crucial for designing effective interventions and policies aimed at achieving health equity.

### 3.1 Human Rights Framework and Global Health Equity

Human rights frameworks provide the essential foundations for addressing global health inequities by establishing legal obligations, normative standards, and accountability mechanisms [55]. The right to health enshrined in international human rights law encompasses both individual entitlements to healthcare services and collective rights to the underlying determinants of health, including clean water, sanitation, food security, and safe environments [55]. Rights‐based approaches to global health equity emphasize several key principles: non‐discrimination and equality, requiring priority attention to the most marginalized populations; participation and empowerment, ensuring meaningful involvement of affected communities in health program design and implementation; accountability and transparency, establishing mechanisms for monitoring state obligations and corporate responsibilities; universality and indivisibility, and recognizing the interconnectedness of civil, political, economic, social, and cultural rights [56]. The application of rights‐based approaches has proven effective in addressing specific health inequities, particularly in the HIV/AIDS response, where human rights frameworks helped combat stigma and discrimination while advocating for treatment access [57]. However, translating rights‐based principles into practice remains challenging, particularly in contexts where power imbalances, resource constraints, and weak governance structures limit the implementation capacity [58]. Historical Injustice and Contemporary Health Inequities Contemporary global health inequities cannot be understood without acknowledging their historical roots in colonialism, slavery, and systematic exploitation. Colonial medicine prioritizes the health needs of colonizers while extracting resources and knowledge from colonized populations, establishing patterns of exploitation that persist in modern global health relationships [59]. The legacy of colonialism continues to shape global health through multiple mechanisms: economic structures that perpetuate resource extraction from low‐income countries; knowledge systems that privilege Western medical paradigms while marginalizing indigenous healing practices; and power dynamics that concentrate decision‐making authority in HICs and international organizations [60]. Decolonizing global health requires the fundamental transformation of power relations, equitable partnership models, and recognition of diverse knowledge systems [60]. This includes addressing the concentration of global health leadership in HICs, supporting locally led health initiatives, and challenging paternalistic approaches that treat low‐income countries as passive recipients of aid rather than as equal partners in global health governance [60, 61].

### 3.2 Real‐World Global Examples

The stark reality of global health inequity is evident in the vast differences in maternal mortality rates between African and Western countries. Limited access to adequate healthcare translates into a disproportionate burden of disease and mortality [7]. Studies have consistently demonstrated inequity in access to healthcare, with poorer access being linked to lower socioeconomic status and other social disadvantages [7]. The inverse equity hypothesis suggests that new interventions initially benefit the wealthy, potentially widening the disparities before reaching the poor [6, 62]. This pattern can be observed in the provision of maternal health services, where coverage of skilled birth attendance has been shown to be significantly higher in wealthier populations than in the poorest ones [8]. Factors such as geographical barriers, financial constraints, and the availability of trained healthcare professionals contribute to these disparities, resulting in tragically higher maternal mortality rates in many African nations than in their Western counterparts [63].

Significant disparities in cancer survival rates also exist between countries, highlighting global health inequities. Socioeconomic inequalities in health can affect health indices across communities and exacerbate poverty and inequality [3]. Global disparities in access to cancer care are a critical concern [3]. Factors, such as the availability of diagnostic tools, treatment options, and healthcare infrastructure, contribute to these differences. HICs often have advanced screening programs and comprehensive treatment facilities, leading to better survival rates compared to LMICs, where access to timely and quality cancer care is limited [64]. These disparities underscore the impact of resource distribution and healthcare system development on health outcomes [16].

The COVID‐19 vaccine rollout vividly illustrates the deep‐seated global health inequities between the North and South. Data from the first year of vaccine deployment revealed dramatic gaps in global access, with HICs achieving significantly higher vaccination rates than low‐income countries [6]. This inequitable distribution was not primarily due to shortages but rather to issues of distribution, with wealthy nations disproportionately receiving the most effective vaccines [6]. This situation reflects a “patent culture” shaped by intellectual property law that can neglect health equity principles, favoring the wealth and power of those controlling vaccine production and supply [6]. The policy choices made by manufacturers and affluent governments contributed to this inequitable‐by‐design vaccination program, falling into the inverse equity pattern of early access for the wealthy, followed by lagging access for the poorest countries [6]. Concerted global efforts are necessary to address such health inequities and foster trust in institutions and health programs to prepare for future public health emergencies [10]. The allocation of market and political power in healthcare systems can exacerbate these inequities, highlighting the need for a re‐evaluation of global health governance and a shift toward principles of public value, transparency, and inclusivity in access to life‐saving treatments [6].

### 3.3 Impacts of Inequality and Inequity

#### 3.3.1 Mortality and Disease Burden

Health inequity results in increased mortality, morbidity, and limitations in functioning in persons with disabilities compared to the general population [14]. Many poor health outcomes experienced by people with disabilities are driven by unfair societal and health system factors, not just by underlying conditions [14]. Avoidable deaths from causes amenable to good quality healthcare are more common in people with intellectual disabilities (37%) than in the general population (13%) in England and Wales, illustrating their impact on mortality [14].

Globally, the health burden caused by Chronic Obstructive Pulmonary Disease (COPD) has increased by 25.7% in terms of disability‐adjusted life years (DALY) from 1990 to 2019 [46]. The burden of COPD is disproportionately concentrated in populations living in countries with low socio‐demographic indices (SDIs) [46]. Socioeconomic conditions are a major determinant of negative outcomes for COVID‐19 patients, regardless of other risk factors [54]. A Brazilian study showed a 32% increase in COVID‐19 mortality among socially vulnerable individuals compared with those with better socioeconomic status [54]. This finding highlights how inequality exacerbates the burden of disease and mortality during public health emergencies.

Evidence suggests that most illnesses and health inequalities stem from social factors [65]. The inverse care law further compounded this, where the worst‐off and least healthy underutilized health services [8, 49]. Socioeconomically disadvantaged groups often have poorer access to health care and adopt effective services later, contributing to a higher disease burden [8, 49].

In older adults in China and Ghana, socioeconomic factors are determinants of inequalities and inequities in health, affecting both single and multiple NCD morbidity [66]. In LMICs, the health of older people is further compromised by poverty and limited access to affordable healthcare, which increases the burden of NCDs [66]. The NCD burden is relatively higher in older age and among disadvantaged groups than in those with higher socioeconomic status [66].

#### 3.3.2 Life Expectancy Gaps

Global health inequality is evident in the significant differences in the average life expectancy between countries. For instance, in 2015, the average life expectancy in Japan was 83.7 years, whereas in Sierra Leone, it was just 50.1 years [49]. Inequalities also exist within countries, with a 20‐year gap in male life expectancy between the richest and poorest areas of Glasgow [49]. Similarly, in Baltimore and Washington, DC, individuals in poor areas have a life expectancy 20 years shorter than those in rich areas [49]. The failure of initiatives such as COVAX to ensure equitable vaccine distribution during the COVID‐19 pandemic has perpetuated the gap in health and well‐being between HICs and LMICs [10].

#### 3.3.3 Economic and Social Consequences

Global vaccine inequity not only causes devastating health impacts but also has a profound impact on socioeconomic recovery in LMICs [10]. The failure of initiatives such as COVAX to ensure equitable vaccine distribution during the COVID‐19 pandemic highlighted how global health inequity can have a lasting and profound impact on socioeconomic recovery in LMICs [10]. Socioeconomic inequalities in health can exacerbate poverty and social inequalities [67]. The significant global burden of diseases such as cancer also has a substantial economic impact, with estimated costs running into trillions of dollars [64].

#### 3.3.4 Intergenerational Cycles of Poor Health

Evidence suggests that income inequality experienced early in life can have long‐lasting negative health implications later in life [45]. Furthermore, low socioeconomic status can result in families, particularly women and children, residing in areas with inadequate health infrastructure, perpetuating a cycle of disadvantage and poor health across generations [68]. Factors such as the poverty experienced by people with disabilities and their limited access to educational opportunities, which impact health literacy, can also contribute to these intergenerational cycles [14].

#### 3.3.5 Recommendations to Tackle Global Health Inequity and Equality

Understanding the root causes of global health disparities is crucial; however, recognizing these issues alone will not lead to meaningful changes unless concrete actions are taken. To equitably improve health outcomes across populations, governments, communities, international organizations, and stakeholders must collaborate to implement targeted strategies that address structural and systemic barriers to health. A multi‐sectoral approach is essential to reducing health inequalities and promoting fairness in access to care. The following interventions offer practical pathways to bridge this health gap and support sustainable improvements in global health equity (Table 1).

**TABLE 1:** Recommendations to tackle global health inequality and inequity.

Solution category	Specific interventions	Implementation actors	Accountability mechanisms	References
**Rights‐based policy frameworks**	Implement human rights‐based approaches to health policy; establish legal frameworks guaranteeing right to health	National governments, WHO, UN human rights bodies	International human rights monitoring (e.g., UN Human Rights Council); domestic constitutional courts; civil society advocacy	[69]
**Commercial determinants regulation**	Regulate harmful industry practices (tobacco, alcohol, unhealthy commodities); implement tobacco control measures; address intellectual property barriers to medicine access	National governments, WTO, pharmaceutical companies	WHO Framework Convention on Tobacco Control (FCTC) monitoring; WTO dispute resolution; civil society watchdogs (e.g., Médecins Sans Frontières)	[70, 71]
**Structural inequality interventions**	Universal health coverage (UHC) implementation; progressive taxation; social protection programs	National governments, World Bank, bilateral donors	Sustainable Development Goals (SDG) monitoring framework; health equity audits; participatory social accountability platforms	[72, 73]
**Global health governance reform**	Democratize global health decision‐making; equitable financing mechanisms; strengthen pandemic preparedness	WHO, G20, multilateral organizations	Global health governance scorecards; transparent budget allocation; independent evaluation mechanisms (e.g., Global Health Security Index)	[74, 75]
**Digital health equity initiatives**	Bridge digital divides; ensure inclusive technology design; build digital health literacy	Technology companies, health systems, educational institutions	Digital equity monitoring; accessibility standards compliance; community feedback mechanisms	[76, 77]
**Environmental justice measures**	Address climate change health impacts; implement environmental protections; strengthen resilience in vulnerable communities	National governments, UNFCCC, environmental agencies	Paris agreement monitoring; environmental health indicators; community‐based monitoring	[78, 79, 80]
**Workforce equity strategies**	Ethical health worker recruitment; strengthen health worker education in LMICs; improve working conditions	WHO, bilateral donors, professional associations	WHO health workforce 2030 monitoring; bilateral agreements on ethical recruitment; health worker satisfaction surveys	[81, 82]

Abbreviation: LMICs, low‐ and middle‐income countries.

#### 3.3.6 Limitations and Methodological Considerations

This narrative review had several important limitations that must be acknowledged. The selective literature search strategy, although appropriate for the broad scope of this analysis, may have inadvertently excluded relevant contributions, particularly those published in languages other than English or in specialized journals outside mainstream global health and public health databases. The narrative approach, while enabling the comprehensive synthesis of complex theoretical frameworks, lacks the systematic rigor of formal systematic reviews and meta‐analyses. The rapid evolution of global health challenges, particularly those highlighted by the COVID‐19 pandemic, means that some analyses may reflect conditions that have changed since then. Additionally, the emphasis on structural and systemic factors, although theoretically justified, may underemphasize individual‐ and community‐level interventions that could meaningfully contribute to health equity goals. The scope of this review requires selective engagement with vast literature across multiple disciplines. Future research should employ more systematic approaches to the specific domains identified here, particularly the intersections between commercial determinants, environmental factors, and health equity outcomes in different regional contexts.

## 4 Conclusion

Global health inequality and inequity persist as profound injustices rooted in systemic disparities in power, resources, and access to care. Evidence reveals that marginalized populations—whether in low‐income nations or disadvantaged communities within wealthy states—face disproportionately poorer health outcomes due to structural barriers, discriminatory policies, and geopolitical inequities. The COVID‐19 pandemic exemplified these disparities, with vaccine nationalism and intellectual property restrictions exacerbating the global divide. Addressing these challenges demands more than incremental policy adjustments; it requires a fundamental reorientation of global health governance toward equity, justice, and accountability. By prioritizing the needs of the most vulnerable through redistributive policies, inclusive health systems, and fairer international cooperation, the global community can move closer to realizing the promise of health as a universal human right. The moral and practical urgency of this task cannot be overstated in that equitable health outcomes are not just an ethical imperative but a foundation for a just and sustainable future.

## Author Contributions

Yusuf Hared Abdi conceptualized the study. Yakub Burhan Abdullahi, Sharmake Gaiye Bashir, Mohamed Sharif Abdi, and Naima Ibrahim Ahmed prepared the first draft of this manuscript. All authors have reviewed and approved the final manuscript. All authors contributed to manuscript review and approved the final version of the manuscript.

## Ethics Statement

This study did not involve any human or animal subjects and thus did not require review by an Institutional Review Board (IRB).

## Conflicts of Interest

The authors declare no conflicts of interest.

## Data Availability

Data sharing is not applicable to this article as no datasets were generated or analyzed during the current study.
